# Migrant women’s experiences of HIV prevention and care in Europe: a qualitative evidence synthesis

**DOI:** 10.1016/j.jmh.2026.100435

**Published:** 2026-08-13

**Authors:** Zuzanna Milewska, Alena Kamenshchikova

**Affiliations:** a Department of Health, Ethics and Society, Care and Public Health Research Institute (CAPHRI), Maastricht University, The Netherlands; b Department of Social Medicine, CAPHRI, Maastricht University, The Netherlands; c Department of Medical Microbiology, Infectious Diseases and Infection Prevention, CAPHRI, Maastricht University, The Netherlands

**Keywords:** HIV, Care continuity, Migrant women, HIV prevention, Intersectionality

## Abstract

**Background:**

HIV remains a global challenge, with migrant women disproportionately affected bearing high HIV prevalence and poor outcomes, including reduced care continuity and higher progression to AIDS. Yet, gender-specific HIV-research remains limited. Therefore, this qualitative evidence synthesis (QES) aims to explore how migrant women in the European Union/European Economic Area and the United Kingdom experience HIV care, and what challenges they encounter.

**Method:**

A QES was conducted in accordance with PRISMA guidance. A systematic search of MEDLINE, Embase, Global Health and Web of Science Core Collection was conducted in June 2025, identifying studies published between January 2015 and June 2025, and focusing on the HIV care continuum, including prevention. The findings were interpreted using intersectionality framework.

**Results:**

Twelve studies met the inclusion criteria, predominantly involving African women living in Western Europe. Three analytical themes were formulated focusing on perceptions of HIV risk and prevention practices; navigation of HIV care in receiving countries; and navigation of stigma, relationships and wellbeing. Stigma was established as a central challenge shaping how women perceived their own risk, engaged with the HIV care, and experienced life after diagnosis. Stigma intersected with power imbalances, legal status, sociocultural contexts, and financial insecurities leading to diverse HIV-related practices.

**Conclusion:**

Migrant women's HIV experiences are shaped by intersecting inequities, such as gender, stigma, ethnicity, legal status, and socioeconomic insecurity. Gender-specific, culturally responsive programmes addressing stigma across care stages, including breastfeeding support, are needed. Evidence gaps around retention in care and viral suppression remain, warranting further research.

## 1 Introduction

HIV remains a key global challenge, with migrants being disproportionately affected (UNAIDS, 2023). In 2023, European Centre for Disease Control (ECDC) reported that migrants have a larger HIV burden than the general population in the European Union/European Economic Area (EU/EEA) (ECDC and WHO, 2024). Furthermore, a study by Alvarez-del Arco et al. (2017) reported that over half of migrants living with HIV in Europe acquired HIV after migration, raising concerns about access to prevention measures such as, condoms, pre-exposure prophylaxis (PrEP) and post-exposure prophylaxis (PEP). Overall, a meta-analysis by Segala et al. (2024) showed that migrants living in EU/EEA and the United Kingdom (UK) encounter worse HIV outcomes compared to non-migrant populations.

Intersecting with migration, gender has shown to be an important factor in shaping healthcare experiences. For instance, a quantitative study by Gil-Salmerón et al. (2021) showed that migrant women in the EU/EEA were more likely to report unmet healthcare needs and discrimination within healthcare services compared to migrant men. Inequities in HIV burden may be linked to several challenges that limit access to timely prevention, testing and treatment services. Specifically, research describing barriers to HIV testing among migrant women in Europe, highlighted women’s lack of trust in receiving countries healthcare systems, experiences of stigma, communication challenges and poor socioeconomic conditions (Owusu et al., 2023). Furthermore, intersecting with migration and gender, occupation plays an important role in HIV care experiences. A study conducted in the Netherlands by Peters et al. (2024) highlighted, among sex workers, migrant women were more vulnerable to HIV acquisition and treatment discontinuation than non-migrant women. Therefore, intersecting identities such as migration status and occupation shape unique inequities, positioning some migrant women at higher risk of HIV acquisition and worse HIV outcomes.

Previous literature on HIV and migrant health has identified a range of challenges and facilitators to accessing HIV care and prevention (Gil-Salmerón et al., 2021; Lebano et al., 2020; Owusu et al., 2023). However, gender-specific analyses remain limited, with vulnerabilities related to power imbalance, gender-based violence and exploitation often understudied (Tan and Kuschminder, 2022). Therefore, in this article we aim to synthesise available evidence from qualitative research on migrant women’s experiences of HIV prevention and care in the EU/EEA and the UK. We focus on qualitative research as it tends to engage with research participants as experts of their own experiences, thus allowing us to learn from those experiences when formulating future research agenda.

**Fig. 1:**
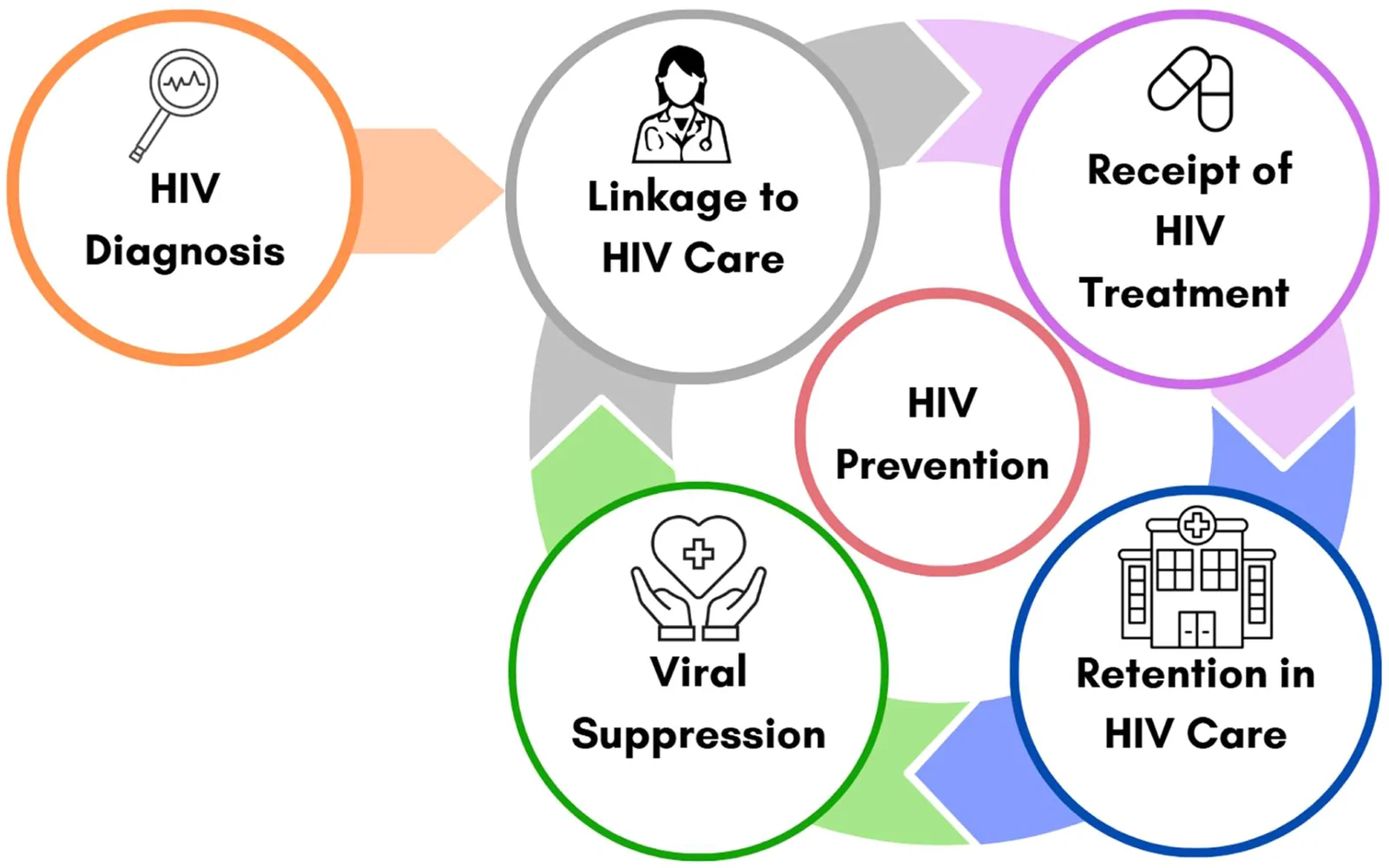
Adapted HIV care continuum, (HIV Care Continuum, 2022).

To define the scope of HIV prevention and care examined in this review, we drew on the HIV Care Continuum (HIV Care Continuum, 2022). It is important to acknowledge that the HIV Care Continuum’s linearity from diagnosis to viral suppression has been criticised for not being able to capture the cyclical journeys of care engagements, particularly among migrants, who may enter and re-enter services as they migrate (Drew et al., 2017; Ehrenkranz et al., 2021). Further, HIV prevention is often not included despite its crucial role in strengthening the HIV response, protecting vulnerable populations and reducing risk of vertical transmission (Huynh et al., 2025). Acknowledging these criticisms, in this review we adapted the continuum, incorporating prevention as a distinct stage and re-conceptualising it as a dynamic and cyclical process to better reflect the non-linear engagements migrant women may experience (Fig. 1).

Understanding migrant women’s experiences requires reflection on the intersecting social identities which may shape HIV acquisition and access to services. Intersectionality, a framework first described by Crenshaw (1989), explains how intersecting systems of oppression such as, racism and sexism create distinct lived experiences. Within health research, this framework has helped analyse inequities that remain hidden in single-axis analyses. For instance, a study by Vohra-Gupta et al. (2023) showed how healthcare access disparities among women are not driven by isolated factors alone but by the interaction between race, socioeconomic status, and marital status, resulting in compounding disadvantages for certain women. In this research, the intersectionality framework informs our interpretation of migrant women’s experiences of HIV prevention and care, and what this means for future public health programmes.

## 2 Methodology

For this review, we followed a qualitative evidence synthesis (QES) methodology, structured using the Cochrane Protocol and Review Template for Qualitative Evidence Synthesis (Glenton et al., 2023). The review followed a pre-determined protocol registered with PROSPERO (registration number: CRD420251080775) and reported in accordance with PRISMA guidance. The adapted HIV Care Continuum defined the relevant stages of HIV care examined in the review, informing the search strategy and the eligibility criteria for included studies. Data was analysed using a thematic approach, informed by deductive and inductive coding. Descriptive and analytical themes were developed, with the intersectionality framework guiding the interpretation of findings.

### 2.1 Selection criteria

The inclusion criteria consisted of published, peer-reviewed primary research articles utilising qualitative methods for data collection and analysis. Mixed-methods studies were included where it was possible to extract data collected and analysed through qualitative methods. Included studies examined migrant women’s experiences of HIV prevention and care in any EU/EEA state or the UK. ‘Migrant’ was defined as someone who changed their country of usual residence for at least three months, irrespective of the reason (Anderson and Blinder, 2024). The review focussed on women assigned female at birth, recognising how reproductive contexts and the risk of vertical transmission shape HIV-related experiences. Studies exclusively involving transwomen were excluded as their HIV-related experiences warrant separate thorough exploration. Eligible studies explored one or more stages of the adapted HIV Care Continuum (Fig. 1). The publication date restriction was the past decade, to reflect the recently increasing number of forced migration (UNHCR, 2025). No exclusions were based on language or methodological quality.

### 2.2 Search strategy

The following databases were searched to identify eligible studies from 1 January 2015 to 8 June 2025 using the search strategies described in Appendix A: MEDLINE; Embase; Global Health; Web of Science Core Collection. All the database searches were conducted in June 2025.

### 2.3 Selection of studies

**Fig. 2:**
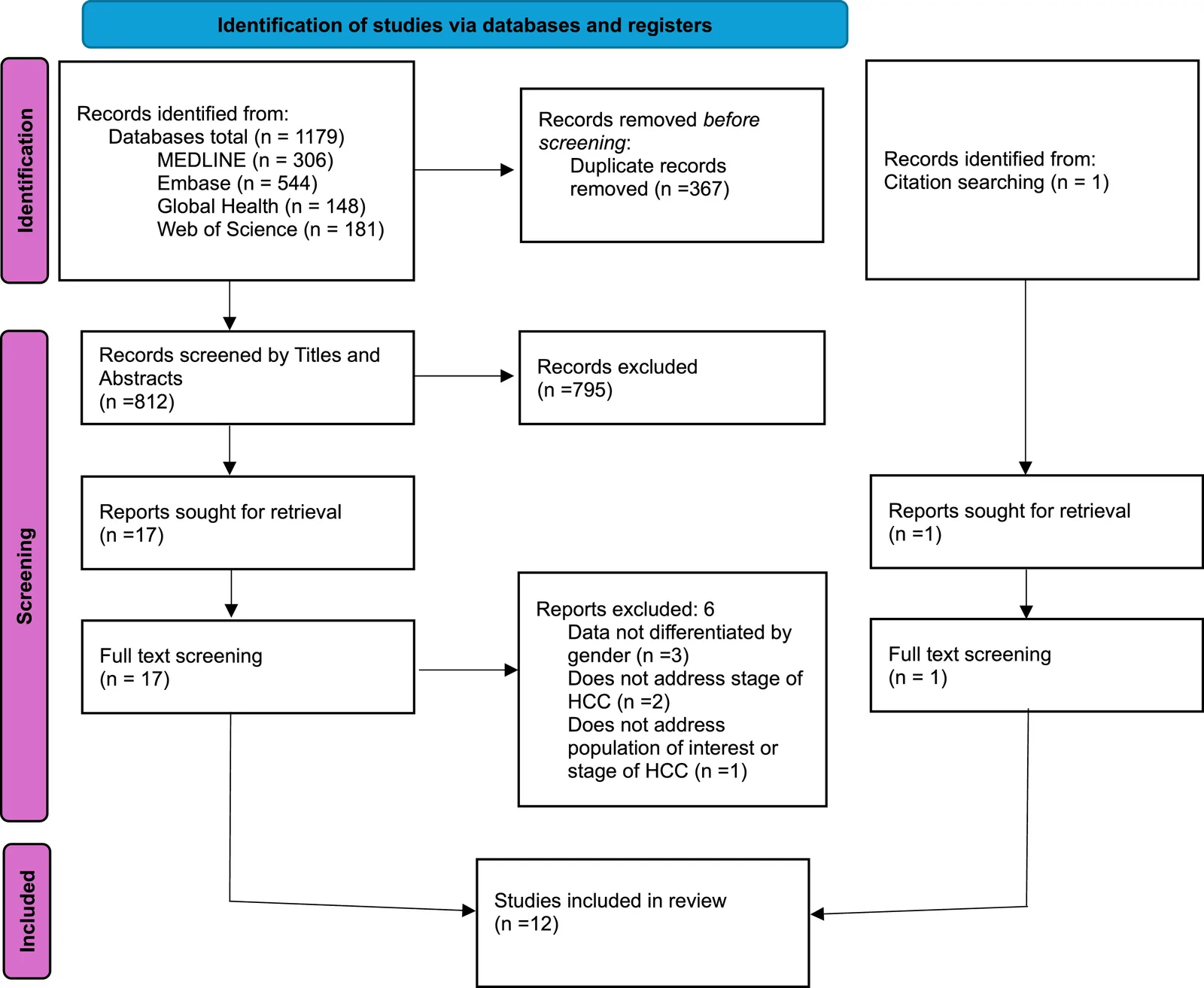
PRISMA flowchart.

Rayyan software was used to remove duplicates and manage selection (Ouzzani et al., 2016). Titles and abstracts retrieved using the search strategy were screened by the first author and any uncertainties discussed with the second author. No language restrictions were applied and where necessary, studies would have been translated for eligibility assessment. However, no study warranted translation. Full texts of potentially eligible studies were assessed by the first author according to the predefined inclusion/exclusion criteria, with uncertainties discussed with the second author. Main reasons for exclusion at full-text assessment were recorded (Appendix B), and bibliographies of included studies were reviewed for additional articles. The study selection process is illustrated by a PRISMA flow diagram (Fig. 2).

### 2.4 Data extraction and synthesis

Full text of included studies was uploaded into ATLAS.ti. The following descriptive study-related data was initially coded (Appendix C): Study author(s); Publication year; Study objectives; Participants; Study location; Intervention (stage/stages of adapted HIV Care Continuum included); Study design (data collection and analysis method). This process was completed by the first author in discussion with the second author.

A thematic approach was used to guide data analysis (Thomas and Harden, 2008), with both deductive and inductive coding (Ayre and McCaffery, 2022). Deductive coding was guided by pre-defined codes derived from the literature review and the research question. Inductive coding was used to generate unanticipated codes from the data. Systematic coding of study findings was conducted by the first author in ATLAS.ti. In mixed-method studies, only qualitative sections were coded. Primary codes were organised into the following descriptive themes: Risk perception; Prevention practices; Experiences with HIV testing, Access to healthcare; Psychosocial wellbeing post diagnosis; Healthcare experiences post-diagnosis; Motherhood post-diagnosis; Adherence to treatment; Stigma and discrimination experiences. After discussion among the authors, the following three main analytical themes were developed: Perception of HIV risk and practices around HIV prevention; Navigating HIV testing and treatment in receiving countries; Navigating stigma, relationships and wellbeing after diagnosis. The intersectionality framework guided the interpretation of the analytical themes, by examining the intersecting social identities, structural factors and migration-related challenges shaping the experiences of migrant women.

### 2.5 Quality assessment

Methodological limitations of included studies were appraised using the Critical Appraisal Skills Programme (CASP) qualitative research checklist (CASP, 2024). This appraisal tool assesses the design, validity and relevance of each study to identify methodological limitations (Appendix D).

Ten studies met >80% of the criteria and were considered to have minor methodological limitations, while two studies had moderate methodological limitations (Appendix D). Majority of studies reported poorly on researcher reflexivity, with only one study stating facilitator positionality (Liegeon et al., 2024). No studies were excluded based on quality assessment as they have included valuable perspectives.

## 3 Results

Overall, twelve studies met the inclusion criteria (Fig. 2), with all studies published between 2015 and 2024. The key characteristics of each included study are summarised in Appendix D. We will first focus on a narrative summary of the study characteristics, then explore the three main analytical themes: Perception of HIV risk and practices around HIV prevention; Navigating HIV testing and treatment in receiving countries; and Navigating stigma, relationships and wellbeing after diagnosis. We used the intersectionality framework to interpret women’s experiences within each theme.

### 3.1 Study characteristics

The twelve included studies used various terminology to sub-categorise migrant women including HIV status, ethnicity, country of birth, pregnancy status, legal status, occupation and sexuality. The heterogeneity of descriptions used reflects how diverse migrant women are, as well as what authors of each study deemed were important social identities influencing women’s experiences. However, this heterogeneity complicates cross-study comparison as sub-categories are not consistent or mutually exclusive.

Five studies focused on HIV prevention (Åkerman et al., 2017; Auli et al., 2015; Di Giuseppe et al., 2019; Liegeon et al., 2024; Tokar et al., 2020), six on women living with HIV (Arrey et al., 2015, 2015ba, 2016a, 2016b; Tariq et al., 2016; Vitale and Ryde, 2018) and one included both (Nakasone et al., 2020). Black African migrant women, predominantly from sub-Saharan Africa (SSA), the region with the highest HIV burden among women and girls (UNAIDS, 2023), were participants in ten studies (Arrey et al., 2015, 2015ba, 2016a, 2016b; Auli et al., 2015; Di Giuseppe et al., 2019; Liegeon et al., 2024; Nakasone et al., 2020; Tariq et al., 2016; Vitale and Ryde, 2018). Legal status was explicitly stated in a minority of studies, despite its known influence on healthcare access (Åkerman et al., 2017; Auli et al., 2015; Tokar et al., 2020; Vitale and Ryde, 2018). Two studies focused specifically on sex workers (Auli et al., 2015; Tokar et al., 2020), and two incorporated additional perspectives from healthcare providers (Arrey et al., 2016a; Tokar et al., 2020).

All studies were conducted in western Europe and seven explicitly set in urban areas (Åkerman et al., 2017; Auli et al., 2015; Di Giuseppe et al., 2019; Liegeon et al., 2024; Nakasone et al., 2020; Tariq et al., 2016; Tokar et al., 2020). All studies addressed at least one stage of the adapted HIV care continuum (Fig. 1), with little focus on retention in care or maintaining viral suppression. The study by Tariq et al. (2016) was unique, examining HIV treatment during and after pregnancy, including prevention of vertical transmission. One study used a mixed-methods research design (Di Giuseppe et al., 2019), while the remaining eleven studies had strictly qualitative designs.

### 3.2 Perception of HIV risk and practices around HIV prevention

Initially, perceiving potential risk of HIV exposure may be required to initiate HIV prevention practices. Throughout the studies, migrant women’s risk perceptions were shaped by their understanding of HIV transmission pathways, occupational and relational risks, and beliefs about the use and effectiveness of prevention measures.

Sex worker migrant women associated higher risk of HIV acquisition with visibly ‘unclean’ individuals (Auli et al., 2015; Tokar et al., 2020). This reinforced the misconception that HIV is associated with physical uncleanliness and therefore, washing or hygiene measures would reduce the risk of HIV acquisition. For example, Nigerian sex workers in Spain described washing after intercourse and douching (Auli et al., 2015), while Eastern European sex workers in Amsterdam described showering before intercourse and inspecting clients hygiene (Tokar et al., 2020) as a way to prevent HIV. These misconceptions that associate HIV with cleanliness, mentioned among sex workers, suggest that occupational exposure combined with limited access to sexual health education may have shaped these specific perceptions regarding a potential risk of HIV exposure.

Comparable misconceptions regarding HIV acquisition were shared by non-sex worker migrant women in Sweden and France who attributed HIV risk to practices such as socialising or sharing objects (Åkerman et al., 2017; Liegeon et al., 2024). For instance, a SSA woman from France described her association of HIV transmission with sharing objects, such as a hair comb:

“HIV isn’t always transmitted by sex, sometimes by an object, when you share a comb with someone” (Liegeon et al., 2024).

In another study, Black African women in the UK viewed HIV as a universal threat to everyone and were not familiar with when to implement preventative measures (Di Giuseppe et al., 2019). These beliefs may reflect a persistent gap in familiarity with transmission pathways which may be shaped by limited targeted sexual education.

Women across multiple studies mentioned infidelity as a key vulnerability to HIV acquisition, perceiving higher risk when infidelity was present in relationships (Åkerman et al., 2017; Arrey et al., 2015a, 2015b; Auli et al., 2015). For instance, a SSA woman from Belgium highlighted the association she had between HIV, marriage and morality:

“HIV is for those who are not married or are unfaithful” (Arrey et al., 2015b).

Interestingly, sex workers in Amsterdam perceived lower risk during sexual encounters with married clients compared to single clients (Tokar et al., 2020), reflecting how the same association between HIV and infidelity was found in both intimate relationships and occupational settings. However, such association may perpetuate stigma that associates HIV with infidelity and reduces women’s perceived need to engage in prevention or testing.

In contrast, other participants showed familiarity with HIV transmission and ways of prevention. For instance, Thai women living in Sweden perceived themselves at lower HIV risk due to trust in their own and their communities knowledge of HIV transmission and condom use (Åkerman et al., 2017). Education about prevention methods was recognised as important by SSA women in France (Liegeon et al., 2024) and Eastern European women in the Netherlands (Tokar et al., 2020). The findings point to high heterogeneity among practices and beliefs among migrant women and thus the need for targeted research and interventions for these different groups.

Across multiple studies, women highlighted condoms as the most common and effective HIV prevention method (Åkerman et al., 2017; Auli et al., 2015; Liegeon et al., 2024; Nakasone et al., 2020). Nigerian sex workers in Spain were motivated to use condoms to protect themselves and intimate partners outside work (Auli et al., 2015). However, both sex workers in Spain and the Netherlands described commonly not using condoms during oral intercourse, and highlighted external pressures, such as higher payments, to sacrifice condom use during sexual intercourse (Auli et al., 2015; Tokar et al., 2020). SSA women living with HIV reported no condom use with partners despite known risk of transmission, due to fear of disclosing their HIV status, desire for pregnancy or yielding to male preference (Arrey et al., 2015b). Across studied contexts, male reluctance to use condoms was consistently reported (Arrey et al., 2015a, 2015b; Auli et al., 2015; Di Giuseppe et al., 2019; Liegeon et al., 2024; Tokar et al., 2020). For example, a SSA woman in Belgium living with HIV described her husband refusing condom use:

“I proposed using condoms but he refused” (Arrey et al., 2015b)

Similarly, an Eastern European sex worker in the Netherlands reported a non-consensual removal of a condom by a client:

“During sexual intercourse he removed his condom” (Tokar et al., 2020).

These two accounts illustrate how gendered power dynamics, relationship expectations, HIV-related stigma, financial incentives and occupational pressures, may limit a women’s ability to negotiate and adopt safer sex practices.

Familiarity with PrEP among migrant women was limited in the three studies addressing it, however, interest was present once the purpose of PrEP was explained (Di Giuseppe et al., 2019; Liegeon et al., 2024; Nakasone et al., 2020). Specifically, Black African women in the UK viewed PrEP as an alternative to condoms and an empowerment tool improving women’s autonomy in HIV prevention (Di Giuseppe et al., 2019). Discussing potential PrEP campaigns, women from the UK and France suggested peer-modelled and tailored education that could be facilitated through healthcare providers, online platforms and media campaigns (Di Giuseppe et al., 2019; Liegeon et al., 2024; Nakasone et al., 2020). Confidentiality was seen as a priority, with women preferring advice and prescriptions to come from trusted healthcare sources (Liegeon et al., 2024; Nakasone et al., 2020).

Multiple challenges hindering PrEP uptake were described. Stigma was a recurring topic in all three studies, where women anticipated partner and community judgement. For instance, a Black African woman in the UK explained how taking PrEP would be misinterpreted as having HIV:

“Words will go around and then they will not look at [PrEP] as prevention, but you have got HIV” (Di Giuseppe et al., 2019).

Additionally, SSA women in France suggested misconceptions linking PrEP with infidelity and concerns of side effects and adherence to a daily pill could hinder uptake (Liegeon et al., 2024).

Together, the findings illustrate that migrant women’s HIV prevention practices are shaped through the intersection of how they perceive risk within relational, occupational and social contexts. While familiarity with HIV transmission routes informed women’s perception of risk, this did not necessarily translate into prevention practices. Prevention practices were constrained by gendered power imbalances, external expectations, financial needs and male dominated sexual decision making. Specifically, for migrant sex workers, financial and occupational pressures can override motivation for condom use. For non-sex workers, gendered relationship expectations, power dynamics, and fear of disclosure have shown to limit condom use. However, it is important to note that such challenges are not unique to migrant women and have been also described by non-migrant women (Fitzgerald et al., 2023).

### 3.3 Navigating HIV testing and treatment in receiving countries

Access to HIV testing varied across settings. For instance, sex workers in Spain accessed HIV testing in health centres (Auli et al., 2015), while in the Netherlands, sex workers in legally licensed venues utilised workplace healthcare outreach (Tokar et al., 2020). However, those working as sex workers in unlicensed venues, online, or independently were often missed from these outreach services (Tokar et al., 2020). For instance, Eastern European sex workers on tourist visas in the Netherlands reported avoiding health services due to fear of deportation, and therefore preferring home-based testing (Tokar et al., 2020). One woman working in the sex industry on a tourist visa in the Netherlands described:

“I don’t want to draw attention…you know, they [health workers] can ask questions” (Tokar et al., 2020).

Additional structural challenges to attend testing centres reported by sex workers in the Netherlands included time constraints and transportation costs (Tokar et al., 2020).

Communication difficulties, unfamiliarity with healthcare systems and cultural norms may hinder access to testing and treatment. For instance, Thai women in Sweden and Eastern European women in the Netherlands both mentioned language barriers and difficulties navigating new healthcare systems led to dependence on others when accessing care and delayed engagement with services (Åkerman et al., 2017; Tokar et al., 2020). One Thai women living in Sweden explained:

“It is a big problem when visiting the healthcare service because you do not get an interpreter” and dependence on partners was apparent with another stating:

“He arranges everything” (Åkerman et al., 2017).

These Thai women relied on their intimate partners for support (Åkerman et al., 2017). However, Eastern European sex workers in the Netherlands often lacked family or friend support and instead relied on pimps or colleagues, which sometimes resulted in a barrier to accessing health services (Tokar et al., 2020). One Eastern European woman in the Netherlands stated:

“ I even do not know how they go to doctors here and where I need to go” (Tokar et al., 2020).

This illustrates how migration status and social context may intersect to create unique vulnerabilities for women, even though both groups required language and navigation support, the relationships that filled it created divergent experiences.

Distrust towards receiving countries’ health systems may have reflected the familiarity with the new system. For instance, Eastern European women in the Netherlands explained that differences between their home and receiving countries’ healthcare systems, such as restricted choice of healthcare provider and lack of out-of-pocket payments, fostered distrust (Tokar et al., 2020). Among SSA women living with HIV, doctors’ recommendations in the UK against breastfeeding conflicted with cultural norms and guidance from home countries, fostering mistrust (Tariq et al., 2016). Despite different reasons for mistrust, both groups preferred returning to their home countries to access healthcare (Åkerman et al., 2017; Tokar et al., 2020).

Following HIV diagnosis, positive patient-provider relationships supported engagement with care (Arrey et al., 2016b; Vitale and Ryde, 2018). For example, refugee women from Africa and Jamaica living in the UK valued continuity of care with one health provider (Vitale and Ryde, 2018). While, SSA women in Belgium stated empathy and non-judgemental care helped normalise lives post-diagnosis and reduced internalised stigma (Arrey et al., 2016b). These examples suggest that positive healthcare relationships could partially offset stigma experienced elsewhere in women’s lives.

Access and adherence to ART were shaped by women’s support networks, acceptance of the condition, religious beliefs and concerns about efficacy and side effects (Arrey et al., 2015a, 2015b, 2016a; Nakasone et al., 2020). ART was viewed by some SSA women as essential to survival (Arrey et al., 2016a) and reducing risk of transmission (Arrey et al., 2015a, 2015b). Supportive partners improved adherence to medication and appointments (Arrey et al., 2015a, 2016a). However, others experienced periods of HIV denial (Arrey et al., 2015b, 2016a), doubted treatment efficacy (Arrey et al., 2015b; Nakasone et al., 2020), raised concerns about visible side effects (Arrey et al., 2015a, 2015b) or were discouraged by religious beliefs (Arrey et al., 2016a). For instance, a SSA women living in Belgium stated:

“All that I fear are the side effects of the medications and the visible signs of the disease” (Arrey et al., 2015a) and another from the same study:

“I was still in denial until the birth of my third child when the doctor told me that I needed to start treatment because my CD4 was very low” (Arrey et al., 2015b).

Legal and socioeconomic constraints may hinder adherence to treatment. For instance, undocumented women in Belgium and France struggled to access ART and healthcare due to difficulty providing proof of residency (Arrey et al., 2016a; Liegeon et al., 2024). An undocumented SSA women in Belgium described being denied ART:

“I was told that I will not be given medications because I was asked to leave Belgium” (Arrey et al., 2016a).

For women experiencing unstable housing or living in poverty, challenges in adherence compounded, with fear of disclosure and financial insecurity preventing safe medication storage and timely medication use (Arrey et al., 2016a).

Overall, these findings highlight how migrant women’s access to and engagement with HIV services is not shaped by legal status alone, but by occupation, structural challenges, socioeconomic status and women’s national, religious and cultural contexts. Precarious legal status and sex work heighten fear of healthcare services as it was often associated with fear of deportation. Reliance on others for access to healthcare produced various outcomes for women in different social and occupational contexts, with some supported by partners and some exploited further by intermediaries.

### 3.4 Navigating stigma, relationships and wellbeing after diagnosis

Quality of life for migrant women living with HIV was shaped by the intersection of relationships, physical symptoms and psychological strain. For instance, women in the reviewed studies described ongoing fear of transmission, stigma and disclosure challenges, while mentioning the importance of support and compassionate care. In multiple studies, women living with HIV mentioned how diagnosis negatively affected their quality of life, with symptoms and opportunistic infections contributing to psychological strain (Arrey et al., 2015a, 2015b, 2016a; Vitale and Ryde, 2018). The unpredictability of HIV symptoms was described by a refugee woman living in the UK:

“My days aren’t predictable. I can get up this morning and I just felt fine and I can wake up tomorrow morning and feel quite sick” (Vitale and Ryde, 2018).

Additionally, refugee women living in the UK reported intersecting challenges of financial instability and housing insecurity alongside dealing with physical symptoms (Vitale and Ryde, 2018).

Fear of transmission intersected with gender and reproductive expectations. Among SSA women in Belgium and Black African and Caribbean women in the UK, fear of transmitting HIV to intimate partners and unborn children shaped intimate relations and family planning (Arrey et al., 2015b; Nakasone et al., 2020). A SSA woman living with HIV in Belgium described:

“I did not have normal sexuality after HIV-positive diagnosis because of fear. I was not sure I wouldn’t infect the babies I had really wanted to have” (Arrey et al., 2015b).

For SSA migrant women living with HIV in Belgium and the UK, fear of transmission intersected with cultural breastfeeding expectations and fear of disclosure (Arrey et al., 2016b; Tariq et al., 2016). Formula-feeding risked unwanted disclosure among their communities, illustrating how cultural context can affect how women navigate healthcare guidance.

Furthermore, some women experienced challenging or abusive reactions from intimate partners following HIV disclosure. Disclosure sometimes led to rejection and blame (Arrey et al., 2015a, 2015b; Vitale and Ryde, 2018), and for some, escalated to emotional or physical violence (Arrey et al., 2015a). Among SSA women in Belgium, partners fear of acquiring the virus disrupted intimacy (Arrey et al., 2015b). These experiences had lasting impacts on women, with some avoiding relationships completely.

Self-stigma compounded partner stigma among African and Jamaican women living with HIV in Belgium and the UK, where internalised cultural and religious narratives associated HIV with infidelity and promiscuity (Arrey et al., 2015b; Vitale and Ryde, 2018). For instance, prior to migration, women from Africa and Jamaica witnessed people diagnosed with HIV being rejected (Vitale and Ryde, 2018), untreated (Liegeon et al., 2024) or dying (Arrey et al., 2016a). These experiences seemed to lead to the internalisation of shame and fear, with some believing they would suffer the same fate as those they had seen exiled and dying from HIV (Arrey et al., 2016a).

Community stigma largely shaped migrant women’s lives, with sex and HIV remaining a taboo among African women in Belgium and the UK (Arrey et al., 2015a; Di Giuseppe et al., 2019). Community HIV-related stigma reported in the UK made some women feel the need to relocate to avoid judgments (Vitale and Ryde, 2018). Fear of being seen at HIV clinics or discussing HIV, further discouraged engagement with services for African and Caribbean women in the UK (Di Giuseppe et al., 2019; Nakasone et al., 2020). In two studies from Belgium and the UK, women linked persistence of stigma to widespread misconceptions and gaps in knowledge about HIV among health providers and the general public (Arrey et al., 2016b; Vitale and Ryde, 2018). A refugee woman living in the UK described a discriminatory encounter with a member of the police force:

“I did tell her I was HIV positive, and she looked at me, she threw down the pen and she said you’re HIV positive? Do you have AIDs?” (Vitale and Ryde, 2018).

Stigma was perceived by migrant women in the UK’s and Spain’s healthcare systems, where women anticipated or experienced HIV-related discrimination from their health providers (Auli et al., 2015; Vitale and Ryde, 2018). African and Jamaican women living with HIV in Belgium and the UK, described health providers showing visible fear towards them and taking excessive precautions when attending to them (Arrey et al., 2015a, 2016b; Vitale and Ryde, 2018). For example, a refugee woman in the UK explained how she was seen last at the dentist after disclosing her HIV diagnosis:

“I waited was up to almost five hours. She (the dentist) could not touch me” and “It took time and I was the last one (in the waiting room)” (Vitale and Ryde, 2018).

A SSA woman from Belgium described experiencing inequitable treatment after HIV disclosure:

“When I tell the service provider that I am HIV positive, he/she starts looking at you differently. One nurse told me that I could not be operated upon before the others because of my HIV-positive status” (Arrey et al., 2016b).

Other women in the same study from Belgium were refused healthcare or accused of promiscuity. Such discriminatory experiences can lead to overall avoidance of care.

Discrimination was multidimensional, with women reporting HIV-related stigma intersecting with discrimination related to their migration status (Arrey et al., 2016b; Vitale and Ryde, 2018), occupation (Tokar et al., 2020), and ethnicity (Nakasone et al., 2020). For example, African and Caribbean women in the UK believed racism and HIV diagnosis compounded to delayed or substandard healthcare (Nakasone et al., 2020). In another study, refugee women living with HIV in the UK felt that post-Brexit xenophobia intensified and compounded their isolation (Vitale and Ryde, 2018). Overlapping and cumulative stigma led to some women living with HIV concealing their diagnosis (Arrey et al., 2015a), avoiding local healthcare services (Arrey et al., 2016b), hiding ART medications (Arrey et al., 2015a, 2016a) and isolating themselves (Arrey et al., 2015a, 2015b).

Despite stigma and multidimensional discrimination, when mitigated, women living with HIV valued emotional support from partners and family (Arrey et al., 2015a, 2015b), psychosocial support from healthcare providers (Vitale and Ryde, 2018) and faith (Arrey et al., 2016a). These findings highlighted the role of compassion and support networks in coping with stigma, building resilience and improving overall wellbeing.

## 4 Discussion

Responding to the growing HIV burden and worse health outcomes among migrant women in EU/EEA and the UK (ECDC and WHO, 2024; Segala et al., 2024; UNAIDS, 2023), in this research we used QES to synthesise migrant women’s experiences of HIV prevention and care. While defining the scope of our review with the adapted HIV Care Continuum, our findings show that current literature has limited focus on retention in care and maintaining viral suppression, despite migrants facing heightened rates of treatment discontinuation and progression to AIDS (Segala et al., 2024). Using an intersectionality framework during data interpretation we reflected on the heterogeneity of experiences among migrant women among included studies (Kapilashrami and Hankivsky, 2018).

Perceiving risk of HIV acquisition was shaped by the intersection of occupational risk exposure and HIV-related stigma rather than knowledge of transmission pathways alone. Specifically, among migrant sex workers in Spain and the Netherlands, occupational exposure with limited sexual health education, produced hygiene-based misconceptions about HIV transmission. Therefore, improving risk perception should start by tackling misconceptions about HIV and stigma among communities and health providers to reduce this perpetuating cycle.

HIV prevention practices are rarely individual choices but are rather outcomes of intersecting occupational, gendered and socio-economic contexts. As we saw from the examples of sex workers, their clients’ preferences, financial incentives and occupational norms compounded. Across included studies male-dominated sexual decision-making limited women’s ability to negotiate condom use, supporting previous literature discussing condom use challenges among women (East et al., 2011; George et al., 2016). Despite recent advancements in biomedical HIV prevention strategies (Mody et al., 2024), non-evidence-based hygiene practices remained popular among sex worker populations included in the reviewed studies.

The topic of PrEP was limited in the analysed studies, and it was shown that research participants were not familiar with the concept. Women in the UK defining PrEP as an empowerment tool reflected broader arguments regarding the potential shift in HIV prevention from being male-dependent to woman-controlled (Murphy et al., 2022). However, anticipated partner and community stigma, alongside other usage concerns, suggest PrEP’s empowerment potential can be constrained. These findings suggest current efforts to utilise PrEP may be inadequately reaching migrant women described in this review, as well as, insufficiently addressing challenges migrant women face to benefit from PrEP. However, further research is required to understand how PrEP is used and perceived across different contexts and populations.

Migrant women’s engagement with healthcare was shaped not only by service availability but by its accessibility, cultural appropriateness and trustworthiness. Specifically, distrust in the Dutch health system by Eastern European women can be seen as rooted in unfamiliarity with this system, while mistrust in the UK’s health system by African women arose from certain cultural norms conflicting doctors’ recommendations. Lack of language interpreters and difficulty navigating health systems, unstable housing and undocumented status’ remained major challenges to consistent care. Continuity and empathy in patient–provider relationships emerged as critical facilitators of engagement, supporting wider evidence that trusted, long-term provider relationships encourage ART adherence and reduce HIV-related stigma (Dawson-Rose et al., 2016; Hassan and Coon, 2024). These findings highlight how structural, legal and relational factors intersect differently across contexts shaping access to health services, underscoring the importance of context-specific approaches to care.

It is important to highlight that life with HIV as reported in the reviewed studies was constructed by the intersection of intimate relationships, community context, healthcare experiences and psychological strain rather than the disease burden alone. Specifically, among SSA women in Belgium fear of transmitting HIV reshaped intimacy and reproductive decisions. Fear among women even when virally supressed, indicates that treatment as a prevention principle, such as undetectable=untransmissible, are not fully embedded in migrant women’s experiences. Stigma was present across multiple contexts, internalised, community and healthcare driven. Migrant women in Belgium, Spain and the UK anticipated or experienced discrimination due to their HIV diagnosis. Discrimination was multifactorial when women experienced intersecting forms of marginalisation related to their legal status, ethnicity and socioeconomic background.

Overall, this review highlights stigma as a central challenge shaping migrant women’s experiences of HIV prevention and care. Persistence of stigma compounded with gender, legal status, ethnicity, occupation, socioeconomic position and cultural context, created layered vulnerabilities to HIV acquisition and challenges with access to HIV prevention and care. While the HIV Care Continuum has been useful in assessing gaps in care, it neglects equally vital psychosocial dimensions of care continuity. Scholars have therefore suggested a “social care continuum” (Rhodes et al., 2019), emphasising HIV care must extend beyond biomedical treatments. For migrant women represented in this review, comprehensive care models require addressing legal and gender-specific vulnerabilities, multifactorial discrimination, alongside offering psychosocial support. However, as these findings are drawn predominantly from African women in Western European countries, they should be interpreted with caution when applied to other migrant women in different contexts. Important to note, currently ongoing reductions in international funding for HIV programmes, especially those historically supported by the U.S., threaten addressing these challenges (UNAIDS, 2025). This funding instability may disrupt access to prevention, testing, and treatment services for vulnerable populations, including the migrant women described in this review.

### 4.1 Strengths and limitations

To our knowledge, this is the first QES collating migrant women’s experiences of prevention and care in the EU/EEA and the UK. Although this review used comprehensive database searches and provided important insights, limitations must be acknowledged. First, the synthesis relied on data analysed and reported by primary authors, which may not fully capture the original raw data. Second, the focus on EU/EEA and the UK, as well as the limited number of countries, health systems and migrant groups represented, reduces generalisability of the findings. Third, by including only published, peer-reviewed studies, the review could present dissemination bias, missing perspectives from marginalised or under-represented groups. Fourth, primary coding and thematic analysis were conducted by a single reviewer, despite discussion with the second author, it may have influenced interpretive reflexivity.

## 5 Conclusion

Despite progress in addressing HIV globally, health inequities persist and continue to shape migrant women’s experiences. Gender, stigma, ethnicity, socioeconomic status, occupation, and legal status intersect to create unique experiences described by migrant women represented in this review. These findings point to the need for gender-specific and culturally responsive HIV programmes that address stigma across all stages of HIV care. Breastfeeding is one such area where international guidelines, such as those by the European AIDS Clinical Society (2025), support breastfeeding for virally suppressed women but are not always supported by healthcare providers. Our findings highlight a gap in the evidence base regarding retention in care and viral suppression among migrant women. Further research is required to explore the experiences of diverse migrant women living with HIV across the EU/EEA countries outside of Western Europe.

## Ethics declaration

The authors have NOT obtained informed consent from participants or their legal representatives. Not applicable as this is a literature review study.

This study was performed in compliance with relevant laws, regulatory frameworks and guidelines where the research took place. Ethics committee approval was not required under relevant laws and institutional guidelines. This study did not require formal ethical approval as only secondary publicly available data was included in the review. No identifiable original data was used.

## CRediT authorship contribution statement

**Zuzanna Milewska:** Writing – review & editing, Writing – original draft, Project administration, Methodology, Investigation, Formal analysis, Data curation, Conceptualization. **Alena Kamenshchikova:** Writing – review & editing, Supervision, Methodology, Conceptualization.

## Declaration of competing interest

The authors declare that they have no known competing financial interests or personal relationships that could have appeared to influence the work reported in this paper.

## Appendix Supplementary materials

Image, application 1
