# Supplementary material for: Migrant women’s experiences of HIV prevention and care in Europe: a qualitative evidence synthesis
Source: J Migr Health. 2026 Aug 13;14:100435. doi: 10.1016/j.jmh.2026.100435 (PMC13524862; doi:10.1016/j.jmh.2026.100435)
Supplement: Supplementary file 1 [file mmc1.docx]

## **Appendices**

### **Appendix A. Search history**

**A.1 Search strategy for Ovid MEDLINE:**

| **Keywords** | **Search string** |
| --- | --- |
| HIV care | 1. exp Anti-HIV Agents/ or exp HIV Infections/ or exp HIV/ 2. (HIV or "Human Immunodeficiency Virus" or AIDS or "acquired immunodeficiency syndrome").mp. 3. 1 or 2 4. exp Patient Care Management/or exp “Quality of Health Care”/ or Universal Health Care/ or “health care quality, access, and evaluation"/ 5. (healthcare or service or medical or care).mp. 6. exp preventive health services/ or exp women's health services/ 7. prevent* .mp. 8. exp Diagnosis/ 9. diagnos*.mp. 10. exp Attitude to Health/ 11. "link*".mp. 12. exp therapeutics/ 13. (receipt or treatment* or manag* or drug* or medication* or therap*).mp. 14. (retention or adhere* or continu* or suppress*).mp. 15. 4 or 5 or 6 or 7 or 8 or 9 or 10 or 11 or 12 or 13 or 14 16. 3 and 15 |
| Migrant women | 17. exp "Transients and Migrants"/ 18. exp Emigrants and Immigrants/ 19. exp refugees/ 20. (migrant* or emigrant* or transient* or foreign* or immigrant* or refugee* or “asylum seeker*”) .mp 21. 17 or 18 or 19 or 20 22. exp Women/ 23. (woman or women or female*).mp. 24. 22 or 23 25. 21 and 24 |
| Experiences | 26. Exp Qualitative research/ or qualitative.mp. 27. “Focus group*”.mp. or Focus Groups/ 28. Exp Interviews as Topic/ 29. interview*.mp. 30. survey*.mp. or exp "Surveys and Questionnaires"/ 31. (experience* or perception* or attitude* or perspective* or barrier* or challenge* or facilitat*).mp. 32. ("mixed method*" or "mixed-method*").mp. 33. 26 or 27 or 28 or 29 or 30 or 31 or 32 |
| EU/EEA | 34. European Union/ or ("European union" or EU or "European economic area*" or EEA).mp. 35. exp austria/ or exp belgium/ or exp bulgaria/ or exp croatia/ or exp czech republic/ or exp hungary/ or exp poland/ or exp romania/ or exp slovakia/ or exp slovenia/ or exp france/ or exp germany/ or exp united kingdom/ or exp greece/ or exp ireland/ or exp italy/ or exp liechtenstein/ or exp luxembourg/ or exp netherlands/ or exp portugal/ or exp "scandinavian and nordic countries"/ or exp spain/or exp cyprus/ or exp malta/ or exp Baltic States/ 36. (Belgium OR Bulgaria OR Cyprus OR Denmark OR Germany OR Estonia OR Finland OR France OR Greece OR Hungary OR Ireland OR Italy OR Croatia OR Latvia OR Lithuania OR Luxembourg OR Malta OR Netherlands OR Austria OR Poland OR Portugal OR Romania OR Slovenia OR Slovakia OR Spain OR Czech Republic OR “United Kingdom” OR UK OR Sweden OR England OR Scotland OR Wales OR “Northern Ireland” OR Iceland OR Liechtenstein OR Norway).mp. 37. 34 or 35 or 36 |
| Combine all concepts | 38. 16 and 25 and 33 and 37 |
| Limit | 39. Limit 38 to ( yr=”2015-current”) |

**A.2 Search Strategy for Embase Ovid:**

| **Keywords** | **Search string** |
| --- | --- |
| HIV care | 1. exp Human immunodeficiency virus/ 2. exp Human immunodeficiency virus infected patient/ or exp Human immunodeficiency virus infection/ 3. exp anti human immunodeficiency virus agent/ 4. (HIV or "Human Immunodeficiency Virus" or AIDS or "acquired immunodeficiency syndrome").mp. 5. 1 or 2 or 3 or 4 6. exp "health care facilities and services" 7. (healthcare or service or medical or care).mp. 8. exp prevention/ 9. prevent* .mp. 10. link*.mp. 11. exp therapy/ 12. (receipt or treatment* or manag* or drug* or medication* or therap*).mp. 13. (retention or adhere* or continu* or suppress*).mp. 14. 6 or 7 or 8 or 9 or 10 or 11 or 12 or 13 15. 5 and 14 |
| Migrant women | 16. exp Migrant/ 17. (migrant* or emigrant* or transient* or foreign* or immigrant* or refugee* or “asylum seeker*”) .mp 18. 16 or 17 19. exp female/ 20. (woman or women or female*).mp. 21. 19 or 20 22. 18 and 21 |
| Experiences | 23. Exp Qualitative research/ or qualitative.mp. 24. Exp interview/ or interview*.mp. 25. survey*.mp. or exp Health Care Survey/ exp health survey/ 26. (experience* or perception* or attitude* or perspective* or "focus group*" or narrative* or barrier* or challenge* or facilitat*).mp. 27. ("mixed method*" or "mixed-method*").mp. 28. 23 or 24 or 25 or 26 or 27 |
| EU/EEA | 29 European Union/ or ("European union" or EU or "European economic area*" or EEA).mp. 30 exp austria/ or exp belgium/ or exp bulgaria/ or exp croatia/ or exp czech republic/ or exp hungary/ or exp poland/ or exp romania/ or exp slovakia/ or exp slovenia/ or exp france/ or exp germany/ or exp united kingdom/ or exp greece/ or exp ireland/ or exp italy/ or exp liechtenstein/ or exp luxembourg/ or exp netherlands/ or exp portugal/ or exp "scandinavian and nordic countries"/ or exp spain/or exp cyprus/ or exp malta/ or exp Baltic States/ 31 (Belgium OR Bulgaria OR Cyprus OR Denmark OR Germany OR Estonia OR Finland OR France OR Greece OR Hungary OR Ireland OR Italy OR Croatia OR Latvia OR Lithuania OR Luxembourg OR Malta OR Netherlands OR Austria OR Poland OR Portugal OR Romania OR Slovenia OR Slovakia OR Spain OR Czech Republic OR “United Kingdom” OR UK OR Sweden OR England OR Scotland OR Wales OR “Northern Ireland” OR Iceland OR Liechtenstein OR Norway).mp. 32 29 or 30 or 31 |
| Combine all concepts | 33 15 and 22 and 28 and 32 |
| Limit | 34 Limit 33 to ( yr=”2015-current”) |

**A.3 Search Strategy for Global Health EBSCOhost:**

| **Keywords** | **Search string** |
| --- | --- |
| HIV care | 1. DE "HIV infections" OR DE "HIV-1 infections" OR DE "HIV-2 infections" OR DE "people living with HIV" OR HIV or "Human Immunodeficiency Virus" or AIDS or "acquired immunodeficiency syndrome" 2. AND DE "health care" OR DE "primary health care" OR DE "tertiary health care" OR DE "disease prevention" OR DE "prophylaxis" OR DE "antiretroviral agents" OR DE "HIV fusion inhibitors" OR DE "HIV integrase inhibitors" OR DE "reverse transcriptase inhibitors" OR healthcare or service or medical or care or prevent* or link* or receipt or treatment* or manag* or drug* or medication* or therap* or retention or adhere* or continu* or suppress* |
| Migrant women | 3. AND migrant* or emigrant* or transient* or foreign* or immigrant* or refugee* or “asylum seeker*” 4. AND DE "women" OR DE "bisexual women" OR DE "employed women" OR DE "heterosexual women" OR DE "homosexual women" OR DE "housewives" OR DE "lactating women" OR DE "pregnant women" OR DE "rural women" or woman or women or female* |
| Experiences | 5. AND DE "qualitative techniques" OR DE "interviews" OR DE "observational studies" OR DE "questionnaires" OR qualitative or interview* or survey* or experience* or perception* or attitude* or perspective* or "focus group*" or narrative* or barrier* or challenge* or facilitat* or "mixed method*" or "mixed-method*" |
| EU/EEA | 6. AND DE "European Union" OR DE "Austria" OR DE "Czech Republic" OR DE "Hungary" OR DE "Poland" OR DE "Slovakia" OR DE "Slovenia" OR DE "Baltic States" OR DE "Estonia" OR DE "Latvia" OR DE "Lithuania" OR DE "Nordic Countries" OR DE "Finland" OR DE "Iceland" OR DE "Scandinavia" OR DE "Cyprus" OR DE "Italy" OR DE "Abruzzi" OR DE "Apulia" OR DE "Basilicata" OR DE "Calabria" OR DE "Campania" OR DE "Emilia Romagna" OR DE "Friuli-Venezia Giulia" OR DE "Italian Marches" OR DE "Latium" OR DE "Liguria" OR DE "Lombardy" OR DE "Molise" OR DE "Piedmont" OR DE "Sardinia" OR DE "Sicily" OR DE "Trentino-Alto Adige" OR DE "Tuscany" OR DE "Umbria" OR DE "Valle d'Aosta" OR DE "Veneto" OR DE "Malta" OR DE "Portugal" OR DE "Azores" OR DE "Madeira" OR DE "Spain" OR DE "Balearic Islands" OR DE "Canary Islands" OR DE "Bulgaria" OR DE "Croatia" OR DE "Greece" OR DE "Crete" OR DE "Rhodes" OR DE "Romania" OR DE "UK" OR DE "Channel Islands" OR DE "Great Britain" OR DE "Isle of Man" OR DE "Northern Ireland" OR DE "Irish Republic" OR DE "Belgium" OR DE "France" OR DE "Alsace" OR DE "Aquitaine" OR DE "Auvergne" OR DE "Brittany" OR DE "Burgundy" OR DE "Central France" OR DE "Champagne Ardennes" OR DE "Corsica" OR DE "Franche Comte" OR DE "Ile de France" OR DE "Languedoc Roussillon" OR DE "Limousin" OR DE "Lorraine" OR DE "Midi Pyrenees" OR DE "Nord Pas de Calais" OR DE "Normandy" OR DE "Pays de la Loire" OR DE "Picardy" OR DE "Poitou Charentes" OR DE "Provence-Alpes-Cote d'Azur" OR DE "Rhone Alpes" OR DE "Germany" OR DE "Baden-Wurttemberg" OR DE "Bavaria" OR DE "Berlin" OR DE "Brandenburg" OR DE "Bremen" OR DE "German Democratic Republic" OR DE "German Federal Republic" OR DE "Hamburg" OR DE "Hesse" OR DE "Lower Saxony" OR DE "Mecklenburg-Pomerania" OR DE "North Rhine-Westphalia" OR DE "Rhineland Palatinate" OR DE "Saarland" OR DE "Saxony" OR DE "Saxony-Anhalt" OR DE "Schleswig-Holstein" OR DE "Thuringia" OR DE "Liechtenstein" OR DE "Luxembourg" OR DE "Netherlands" or "European union" or EU or "European economic area*" or EEA or Belgium OR Bulgaria OR Cyprus OR Denmark OR Germany OR Estonia OR Finland OR France OR Greece OR Hungary OR Ireland OR Italy OR Croatia OR Latvia OR Lithuania OR Luxembourg OR Malta OR Netherlands OR Austria OR Poland OR Portugal OR Romania OR Slovenia OR Slovakia OR Spain OR Czech Republic OR “United Kingdom” OR UK OR Sweden OR England OR Scotland OR Wales OR “Northern Ireland” OR Iceland OR Liechtenstein OR Norway |
| Limit | yr=2015-2025 |

**A.4 Search Strategy for Web of Science Core Collection:**

| **Keywords** | **Search string** |
| --- | --- |
| HIV care | #1 TS=(HIV or "Human Immunodeficiency Virus" or AIDS or "acquired immunodeficiency syndrome")  #2 TS=(healthcare or service or medical or care or prevent* or link* or receipt or treatment* or manag* or drug* or medication* or therap* or retention or adhere* or continu* or suppress*) |
| Migrant women | #3 TS=(migrant* or emigrant* or transient* or foreign* or immigrant* or refugee* or “asylum seeker*”)  #4 TS=(woman or women or female*) |
| Experiences | #5 TS=(qualitative or interview* or survey* or experience* or perception* or attitude* or perspective* or "focus group*" or narrative* or barrier* or challenge* or facilitat* or "mixed method*" or "mixed-method*") |
| EU/EEA | #6 TS=("European union" or EU or "European economic area*" or EEA or Belgium OR Bulgaria OR Cyprus OR Denmark OR Germany OR Estonia OR Finland OR France OR Greece OR Hungary OR Ireland OR Italy OR Croatia OR Latvia OR Lithuania OR Luxembourg OR Malta OR Netherlands OR Austria OR Poland OR Portugal OR Romania OR Slovenia OR Slovakia OR Spain OR Czech Republic OR “United Kingdom” OR UK OR Sweden OR England OR Scotland OR Wales OR “Northern Ireland” OR Iceland OR Liechtenstein OR Norway) |
| Combine all concepts | #7- #6 AND #5 AND #1 AND #2 AND #3 AND #4  Limit – publication date 01-01-2025 to 02-06-2025 |

### **Appendix B. Characteristics of excluded studies**

| Study | Reason for exclusion |
| --- | --- |
| (Glendinning et al., 2019) | Data not differentiated by gender |
| (Kasadha et al., 2025) | Does not address stage of HCC |
| (Nakakawa et al., 2025) | Data not differentiated by gender |
| (Mosnier et al., 2024) | Does not address population of interest and stage of HCC |
| (Rogers & McKeown, 2020) | Data not differentiated by gender |
| (Arrey et al., 2017) | Does not address stage of HCC |

### **Appendix C. Key characteristics of the included studies**

| **Study author** | **Study objectives** | **Participants** | **Location** | **Intervention** | **Study design** |
| --- | --- | --- | --- | --- | --- |
| (Di Giuseppe et al., 2019) | Investigated factors influencing preexposure prophylaxis (PrEP) uptake | - Black African heterosexuals - 247 participants for survey women and men - 18 women for focus groups - Majority born in Africa, heterosexual. - Without HIV diagnosis | East London and Hertfordshire, UK | HIV prevention | Surveys  Focus groups  Thematic analysis |
| (Liegeon et al., 2024) | To explore the knowledge, attitudes, and perspectives toward PrEP | - 19 Sub-Saharan African (SSA) born women - Ages 28-37 - Spoke English or French - Without HIV diagnosis | Paris, France | HIV prevention | Focus groups  Content analysis |
| (Arrey et al., 2015a) | Disclosure of HIV positive status, reasons for disclosure/non-disclosure and how they deal with HIV disclosure. | - 28 sub-Saharan African (SSA) migrant women - Diagnosed with HIV - Receiving HIV/AIDS treatment in Belgium - Spoke French or English - From 14 different countries in SSA - 16/28 higher education, 10/28 secondary education, 1/28 primary education and 1/28 no education | Belgium | HIV prevention, linkage to care and receipt of treatment | Interviews  Thematic analysis |
| (Åkerman et al., 2017) | Explored healthcare-seeking behaviour in relation to sexual and reproductive health and views on HIV prevention | - 19 Thai-born women - Without HIV diagnosis - 24-50 yrs old - Lived in Sweden for less than 5 years - 3/19 university, 10/19 secondary education, 5/19 primary, 1/19 no education - 10/19 employed, 12/19 language studies | Stockholm county, Sweden. | HIV prevention | Interviews  Thematic analysis |
| (Tokar et al., 2020) | Context-specific vulnerabilities, and to describe whether and how these affect their access to HIV testing | 5 Migrant female sex workers (FSWs) from Belarus, Moldova, Russia and Ukraine (Eastern Europe, non-EU countries)   - Without HIV diagnosis - 2/5 had dutch residency, 2/5 toursit visa and 1/5 owner of agency   31 Key stakeholders who worked with migrant sex workers (Government organisations and NGOs )   - Social nurses - Psychologist - Physician - Healthcare provider - Counseller and tester - Director - Researcher - City council rep - Journalist - Social worker | Amsterdam, The Netherlands | HIV testing | Interviews  Observations  Thematic analysis |
| (Arrey et al., 2016b) | This study aimed to investigate stigma and discrimination in health care settings and their effect on the health-seeking behaviour of people living with HIV. | 44 sub-Saharan African migrant women   - Diagnosed with HIV - Spoke English or French - Receiving health care in Belgium - 20-67 yr old | Belgium | Linkage to care | Interviews  Observations  Hospital records  Thematic analysis |
| (Tariq et al., 2016) | Explore, in detail, decision-making about infant feeding among mothers living with HIV. | - 23 migrant African pregnant women recruited - Born in sub-Saharan Africa - Diagnosed with HIV | London, UK | HIV during and after pregnancy- infant feeding decisions due to risk of vertical transmission (HIV prevention). | Interviews  Thematic analysis |
| (Arrey et al., 2016a) | Explores perspectives on HIV treatment/care | 44 Sub-Saharan African (SSA) migrant women   - Diagnosed with HIV - Spoke English or French - Receiving health care in Belgium - 20-67 yr old - Half-finished secondary education, 1/44 had no education | Belgium. | HIV treatment. | Interviews  Observations  hospital records  thematic analysis |
| (Auli et al., 2015) | Determine the risk perception of sexually transmitted infections (STIs) and HIV. | 8 Nigerian commercial sex workers (CSWs)   - Illegal residents - Without HIV diagnoses | Raval area in Barcelona, Spain. | HIV prevention and diagnosis. | Interviews  Interpretative content analysis |
| (Vitale & Ryde, 2018) | Explore specific risk factors affecting the mental health | 8 refugee women, 7 from Africa and 1 from Jamaica.   - Forced migrants (refugee) - Diagnosed with HIV - receiving treatment for their HIV - Spoke English - 30-55 yr old - 6/8 have leave to remain, 2/8 temporary leave status - 7/8 single - 7/8 unemployed, 1/8 employed | Southwest England | Mostly focuses on mental health but also on experiences with HIV care that led to poor mental health- explains how HIV is not very holistic. | Interviews  Thematic analysis |
| (Arrey et al., 2015b) | Examine sexual behaviour | 40 sub-Saharan African (SSA) migrant women   - Diagnosed with HIV - Spoke English or French - Receiving health care in Belgium - 20-67 yr old - Half-finished secondary education - 3/40 retired, 4/40 disabled, 23/40 employed, 10/40 unemployed - 23/40 mothers | Belgium | HIV prevention | Interviews  Observations  Thematic analysis |
| (Nakasone et al., 2020) | This study examined barriers and facilitators to PrEP awareness and candidacy perceptions to help inform PrEP programmes and service development. | 32 Black African/Black Caribbean women   - With and without HIV diagnoses - 19-63 yr old - Sexually active in last 6 months - Born outside the UK - Majority heterosexual | London and Glasgow. | HIV prevention | Interviews  Thematic analysis |

### **Appendix D. Assessment of methodological limitations**

| CASP Criteria  Paper | 1.Was there a clear statement of the aims of the research? | 2. Is a qualitative methodology appropriate? | 3. Was the research design appropriate to address the aims of the research? | 4. Was the recruitment strategy appropriate to the aims of the research? | 5. Was the data collected in a way that addressed the research issue? | 6. Has the relationship between researcher and participants been adequately considered? | 7. Have ethical issues been taken into consideration? | 8. Was the data analysis sufficiently rigorous? | 9. Is there a clear statement of findings? | 10. Is this valuable research? | **Overall assessment of methodological limitations** |
| --- | --- | --- | --- | --- | --- | --- | --- | --- | --- | --- | --- |
| (Di Giuseppe et al., 2019) | Yes | Yes | Yes | Yes | Yes | Cannot tell | Yes | Yes | Yes | Yes | **Minor** |
| (Liegeon et al., 2024) | Yes | Yes | Yes | Yes | Yes | Yes | Yes | Yes | Yes | Yes | **None** |
| (Arrey et al., 2015a) | Yes | Yes | Yes | Yes | Yes | Cannot tell | Yes | Yes | Yes | Yes | **Minor** |
| (Åkerman et al., 2017) | Yes | Yes | Yes | Yes | Yes | Cannot tell | Yes | Yes | Yes | Yes | **Minor** |
| (Tokar et al., 2020) | Yes | Yes | Yes | Yes | Yes | Cannot tell | Yes | Yes | Yes | Yes | **Minor** |
| (Arrey et al., 2016b) | Yes | Yes | Yes | Yes | Yes | Cannot tell | Yes | Yes | Yes | Yes | **Minor** |
| (Tariq et al., 2016) | Yes | Yes | Yes | Yes | Yes | Cannot tell | Yes | Yes | Yes | Yes | **Minor** |
| (Arrey et al., 2016a) | Yes | Yes | Yes | Yes | Yes | Cannot tell | Yes | Yes | Yes | Yes | **Minor** |
| (Auli et al., 2015) | Yes | Yes | Yes | Yes | Yes | Cannot tell | Yes | Yes | Yes | Yes | **Minor** |
| (Vitale & Ryde, 2018) | Yes | Yes | Yes | Cannot tell | Yes | Cannot tell | Yes | Cannot tell | Yes | Yes | **Moderate** |
| (Arrey et al., 2015b) | Yes | Yes | Yes | Cannot tell | Yes | Cannot tell | Yes | Cannot tell | Yes | Yes | **Moderate** |
| (Nakasone et al., 2020) | Yes | Yes | Yes | Yes | Yes | Cannot tell | Yes | Yes | Yes | Yes | **Minor** |
